# Enhancing Veliparib PARP1 inhibitor stability against UVC degradation *via* DPPG liposome encapsulation

**DOI:** 10.1039/d5ra02652k

**Published:** 2026-03-17

**Authors:** Carlota J. F. Conceição, Elin Moe, Paulo A. Ribeiro, Maria Raposo

**Affiliations:** a Laboratory of Instrumentation, Biomedical Engineering and Radiation Physics (LIBPhys-UNL), LA-REAL, Department of Physics, NOVA School of Science and Technology, Universidade NOVA de Lisboa 2829-516 Caparica Portugal mfr@fct.unl.pt; b ITQB NOVA, Instituto de Tecnologia Química e Biológica António Xavier, Universidade Nova de Lisboa 2780-157 Oeiras Portugal; c Department of Chemistry, UiT—The Arctic University of Norway N-9037 Tromsø Norway

## Abstract

Poly(ADP-ribose) polymerase inhibitors (PARPi) are often used in complementary cancer therapy with radiotherapy and chemotherapy, but present some limitations that encapsulation may circumvent. Since conventional PARPi therapy was proven to increase cell sensitization to UV irradiation, and a shower of particles or radiation with energies equal to or below UV can be created during radiotherapy treatments, this work evaluates the effect of UVC on the degradation process and inhibitory capability of Veliparib encapsulated in 1,2-dipalmitoyl-*sn*-glycero-3-phospho-rac-(1′-glycerol) sodium salt (DPPG) liposomes, as well as identifies potential degradation products. Results demonstrate that Veliparib is sensitive to UVC radiation, leading to the degradation of the benzamide ring and carbonyl functional group. This coincides with Veliparib's inhibitory capability loss in PARP1's automodification activity. Furthermore, DPPG encapsulation was shown to protect Veliparib from UVC irradiation until 30 min of exposure. This was translated into a delay in the appearance of a degradation band at 330 nm. Infrared analysis revealed that this band is associated with the degradation of the benzamide ring and the carbonyl functional group. The latter presents several C=O infrared vibrational bands associated with isomerization and degradation of the functional group. The results allowed us to infer the potential degradation process of Veliparib due to UVC irradiation, which is associated with linear unsaturated aldehyde and conjugated ketone structures that arise from the degradation of the benzamide pharmacophore.

## Abstract 2

Evaluation of UVC-induced degradation of Veliparib, a poly(ADP-ribose) polymerase inhibitor, encapsulated in DPPG liposomes and its effect on inhibitory activity, with identification of resulting degradation products.

## Graphical abstract

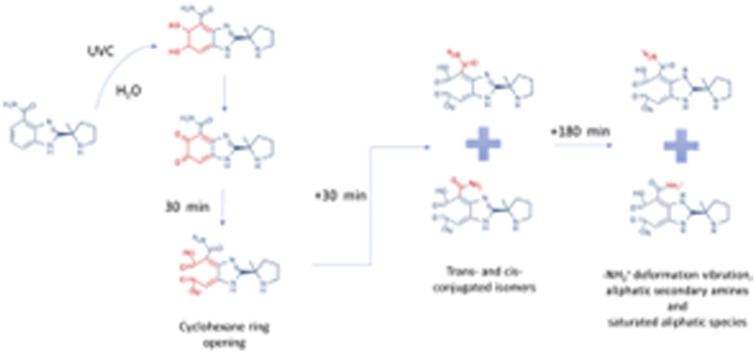

## Introduction

The Poly (ADP-ribose) polymerase (PARP) family, consisting of 17 members, displays a wide range of cellular localizations and functions.^1^ PARP1, a member of the DNA-dependent sub-group, plays direct and/or indirect roles in DNA repair, transcription, replication, cell cycle progression, and other pathways.^2,3^ Its diverse intracellular functions make PARP1 an attractive target for therapeutic approaches.^1–5^ Even more, PARP1 enzymatic activity is hindered by PARP inhibitors (PARPi). By mimicking NAD^+^, these inhibitors can be used alongside conventional cancer treatments.^4,5^ PARPi bind to catalytic domain (CAT) of PARP1, preventing the addition of ADP-ribose polymeric chains (PARylation)to target proteins.^4,6^ These inhibitors take advantage of the compromised DNA repair pathways in cancer cells and prevent the repair of DNA strand breaks. The inhibition of PARP1's auto-PARylation prevents its release from the DNA strand breaks and consequently hinders the allocation of Base Excision Repair (BER) proteins to these loci.^4,6^ In addition, PARPi inhibitors can be used in synergistic lethal strategies, namely, in combinational therapies employing DNA damage agents (*e.g.*, radiation or chemotherapeutics), or as monotherapy agents.^7–9^

Despite their therapeutic potential, inhibitors present several drawbacks,^10,11^ including the development of resistance to therapy, as, for example, the interplay between c-MET membrane receptor and epidermal growth factor receptor-EGFR),^12,13^ cytotoxic side-effects, drug high clearance rates, and even interaction with plasma protein (values > 83% of binding).^10,11^ However, these drawbacks may be circumvented by nano-delivery systems such as liposomes.^14^

Moreover, conventional PARPi therapy was proven to increase cell sensitization to UV irradiation,^15^ and positive feedback was reported when irradiation was combined with PARPi, leading to cancer cell death.^7–9,16–18^ It was verified that even low-wavelength sources, such as UVC light, could activate PARylation due to intracellular DNA damage, and PARP1 inhibition further sensitized cells.^19^ However, information regarding the effect of UVC exposure on the capability of PARPi to retain or lose their therapeutic inhibition on PARP1 enzymatic activity is still lacking. This information is of great importance since cancer therapy's efficacy may be lost or reduced if radiation exposure hinders or reduces the inhibitory capability of therapeutic molecules. Another gap in the literature is associated with the synergistic effect of encapsulating PARP1 inhibitors, for example, in liposomes, and how encapsulation could confer or not protect against radiation. Accordingly, we seek to shed light on the limitations and potential flaws that may arise during cancer treatment, which can compromise clinical compounds' benefits and therapeutic capability. To our knowledge, no studies to date have reported on these topics, including the degradation processes or degradation products of PARPi when exposed to low-energy radiation.

It is also worth emphasizing that one can also take advantage of the opportunity to combine PARPi therapy with radiotherapy. During radiotherapy, ionizing radiation interacts with matter and produces, within femtosecond timescales, numerous ions, radicals, excited neutrals, and ballistic secondary electrons with initial kinetic energies below 100 eV.^20,21^ This cascade of low-energy species produces both physical and chemical alterations in biological media and, consequently, understanding the effects of low-energy UV radiation on inhibitor molecules is of significant interest.

In this work, we aim to analyze the effect of UVC radiation on Veliparib, a PARP1 inhibitor, with particular emphasis on its inhibitory capacity, and to assess the potential protective effect provided by drug encapsulation in liposomes of 1,2-dipalmitoyl-*sn*-glycero-3-phospho-rac-(1′-glycerol) sodium salt (DPPG). For this, the degradation process, products, and inhibitory capacity of Veliparib, encapsulated or not, were characterized. This data is of fundamental importance to ascertain the applicability of DPPG liposomes and conjugated therapy in Veliparib's approach to cancer treatment. This constitutes the first report regarding the effect of low-energy radiation on PARP1 inhibitors encapsulated in liposomes or not. However, it should be noted that we followed the established and optimized protocols for liposomal formulations encapsulating Veliparib, as described in ref. 22, where the variability and reproducibility of these liposomes was also analyzed. Moreover, the encapsulation is due to the interaction which occurs between the protonated amine groups of Veliparib with lipid's carbonyl groups.^22^

## Results and discussion

### Analysis of UV damage on Veliparib and DPPG encapsulating Veliparib *via* UV-vis spectroscopy

**Fig. 1:**
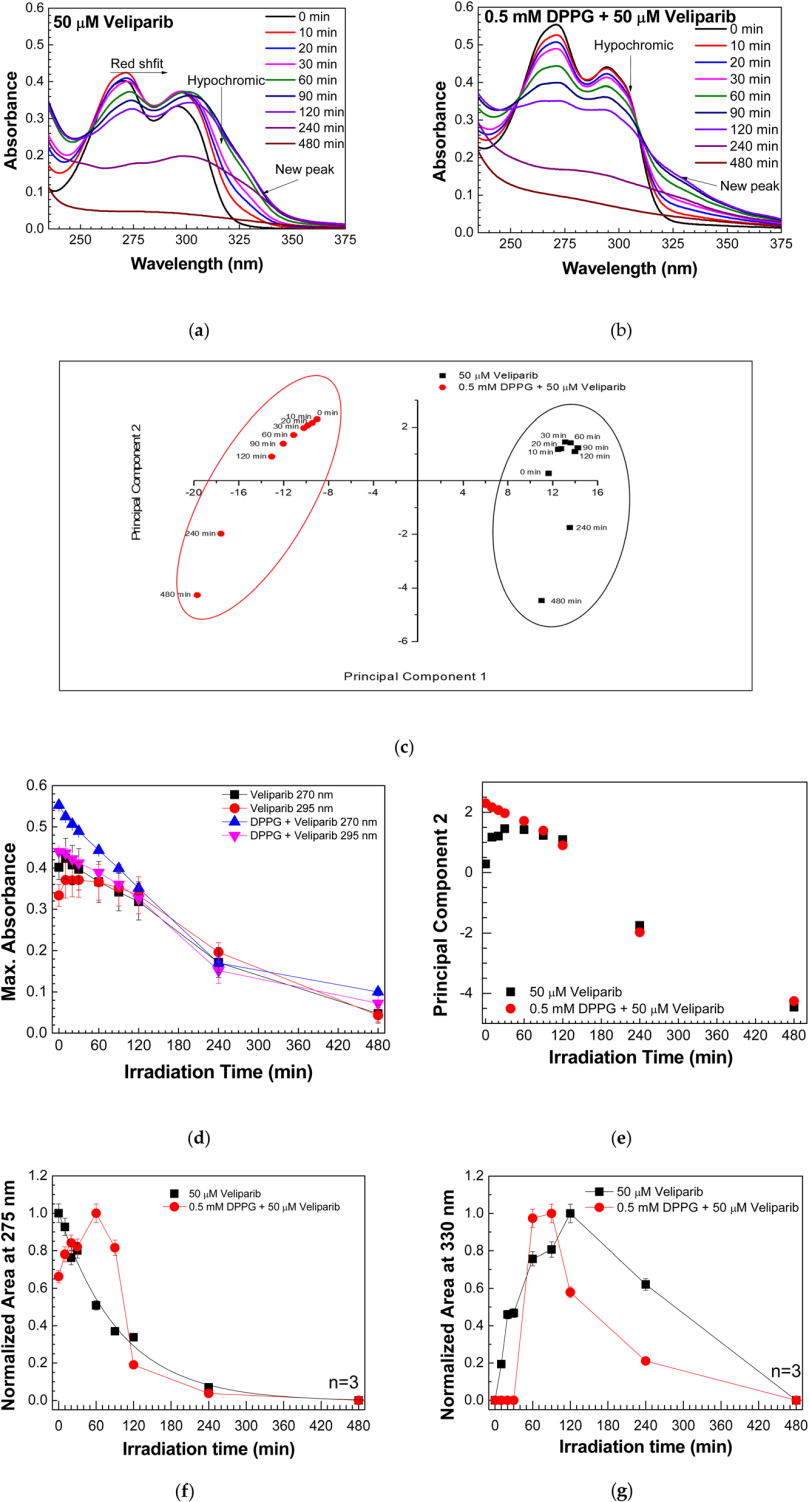
Evaluation of the effect of UVC irradiation on Veliparib and DPPG encapsulating Veliparib. (a) UV-vis spectra of 50 µM Veliparib at different irradiation periods; (b) UV-vis spectra of 0.5 mM DPPG + 50 µM Veliparib at different irradiation times; (c) PCA plot of UV-vis spectra of 50 µM Veliparib and of 0.5 mM DPPG + 50 µM Veliparib solutions at different irradiation times. PC1 and PC2 present 94.816 and 3.264% variance, respectively; (d) analysis of maximum absorbance at 270 nm and 295 nm for Veliparib and DPPG + Veliparib samples at different irradiation times; (e) plot of PC2 values *versus* irradiation time of Veliparib and DPPG + Veliparib samples; (f) analysis of normalized areas of Gaussian 2 (∼275 nm) from irradiated Veliparib and DPPG + Veliparib spectra; (g) analysis of the normalized regions of Gaussian 5 (∼330 nm) from irradiated Veliparib and DPPG + Veliparib spectra. In the graphs, the lines between points are only guidelines.

UV-visible spectroscopy was used to analyze the UVC (254 nm) damage on 50 µM and 0.5 mM DPPG encapsulating 50 µM Veliparib. Absorption spectra of Veliparib (Fig. 1(a)) present two peaks at 270 nm and 295 nm, which are assigned to π–π* transitions from benzene^23^ and imidazole,^24–26^ respectively. The spectra of DPPG liposomes display the typical maximum absorption peak near 194 nm ^27^ (Fig. 5(a), see Annex A), consistent with the characteristic liposome band at 194.4 ± 0.7 nm.^26,28^ This is assigned to the lone-pair transition of carbonyl oxygen to the antibonding π_CO_ valence orbital, n_O_ → π*_CO_, or to the valence shell electronic excitation of hydroxyl groups.^22,28^ The spectra of DPPG encapsulating Veliparib emulsions present the characteristic absorption peaks of the inhibitor, and DMSO band around 205–210 nm (Fig. 1(b) and 5(b)).

Upon irradiation, the samples presented a gradual degradation process (Fig. 1(a), (b) and 5). Liposome formulation presented a new peak around 210 nm, which may be related to lipid peroxidation.^28^ Lipid degradation may occur through two processes: hydrolysis and/or oxidation. Exposure to ionizing irradiation triggers the formation of water radicals (*e.g.* hydroxyl (OH), hydrogen peroxide (H_2_O_2_), and hydroperoxyl), that attack methylene groups from polyunsaturated lipids. The oxidation process can then be monitored by the absorption band of conjugated dienes (215 nm and 250 nm band), resulting from lipid oxidation.^28,29^ In the case of saturated phospholipids, the ester bonds and hydroxyl groups are attacked, and common DPPG degradation products are dipalmitoyl phosphatidic acid, 1,2-dipalmitoyl-*sn*-glycerol-3-phospho-(1,3-dihydroxyacetone), dipalmito-yl-*sn*-glycerol-3-phosphoryl, 1,2-dihydroxypropaldehyde, 1-palmitoyl-snpropanediol-3-phosphorylglycerol and 1,2-dipalmitoyl-snglycerol-3-phosphorylethanol.^28,30^ Owing to the significant DMSO band observed in the DPPG + veliparib samples (Fig. 5(b)), which coincides with the lipid peroxidation region, it is not possible to infer the contribution of the inhibitor to the DPPG degradation process from UV-vis spectra, meaning that it is necessary to study the irradiation effect by infrared spectroscopy.

Veliparib presents an overall change in its UV-vis spectra upon radiation exposure (Fig. 1(a) and (b)). An overall shift to higher wavelengths (redshift) and the appearance of a new absorbance band around 330 nm were observed (Fig. 1(a)). On the other hand, encapsulated Veliparib apparently did not present a redshift of the spectra, but a band around 330 nm also surfaces upon radiation exposure (Fig. 1(b)). The principal component analysis of UV-vis spectra revealed a complete segregation among the irradiated samples, confirming that they behave independently in the irradiation assessment (Fig. 1(c)). Since no significant differences were detected after plotting maximum absorbance values *versus* time of irradiation at 270 nm and 295 nm (Fig. 1(d)), in both samples, a complementary analysis was done decomposing the UV-visible absorbance spectra with Gaussian curves. Additionally, the plot of PCA2 *vs.* irradiation time revealed that differences in the degradation process are detected at initial irradiation time points (Fig. 1(e)).

The analysis of Veliparib's UV-vis spectra (Fig. 6) reveals that these comprise four Gaussians. After DPPG encapsulation, a shift to higher wavelength values is verified (Fig. 6 and Table 1). The shift may be a result of a higher protonation state and/or the presence of a substituent (*e.g.* –OH) in the chromophore of the inhibitor, resultant from the encapsulation and interaction with the liposome membrane.^18^ The data revealed in this work constitute an update to our previous assessments regarding the encapsulation of PARPi in DPPG liposomes. In previous work,^22^ we verified a red shift on the Rucaparib and Niraparib UV-vis spectra after encapsulation, while in Veliparib, this event was not detected. The reason behind the difficulty in verifying the red shift on Veliparib spectra could be due to an overall “masking effect”, since only a major wavelength shift is detected in Gaussian 1 (+5 nm), while the remaining Gaussians present discrete differences (1–2 nm), as depicted in Table 1.

**Table 1:** Gaussian analysis of UV-vis spectra of Veliparib and DPPG + Veliparib samples

Drug Gaussian analysis	Veliparib peak position^a^ (nm)	DPPG + Veliparib peak position^a^ (nm)
1	259.8 ± 0.8	264.9 ± 0.3 (+5)
2	273.6 ± 0.4	275.2 ± 0.1 (+2)
3	292.6 ± 0.2	294.3 ± 0.1 (+2)
4	305.1 ± 0.3	306.3 ± 0.1 (+1)

a Wavelength ± SD.

Spectra deconvolution revealed that the effects of UVC irradiation on Veliparib, either encapsulated or non-encapsulated, are most significant in Gaussian 2 (∼275 nm) and in the band that arises above 300 nm (Fig. 1(f) and (g)).

Analysis of Gaussian 2 reveals that, with irradiation, the wavelength at maximum absorbance, peak position, is roughly stable, shifting from 273.± 0.4 nm to 275.2 ± 0.5 nm and 275.3 ± 0.1 to 278.7 ± 0.6 in Veliparib and DPPG + Veliparib, respectively (Table 2). The analysis of the results revealed that only the normalized area of the absorbance band at 275 nm data for non-encapsulated Veliparib allows us to calculate the characteristic time of degradation, which takes a value of 98 ± 6 min. Analysis of normalized Gaussian areas (Fig. 1(f)) reveals that inhibitor degradation is delayed in DPPG + Veliparib in the initial periods of irradiation (0 to 90 min). One verifies that the area of Gaussian 2 in Veliparib decreases with the exposure to UVC time, while the normalized areas of encapsulated Veliparib appear to be sustained or even increased until 90 min of irradiation (Fig. 1(f)). After 90 min of irradiation, a decay characteristic time value of 56 ± 5 min is observed, indicating that the decay process is faster than the one observed on non-encapsulated veliparib. In both samples, Gaussian 2 disappears completely after 480 min of UVC exposure, which translates into complete compound degradation (Fig. 1(a) and (b)).

The detection of the new absorbance band after UVC exposure can be hypothesized as related to a degradation product of Veliparib. The wavelength at maximum absorbance of this product (Gaussian 5) shifts from 325.2 ± 0.8 nm to 330.3 ± 0.9 nm and 317.2 ± 3.1 nm to 331.3 ± 0.4 nm in Veliparib and DPPG + Veliparib, respectively (see Table 2). But more interestingly, we verified that the surface of this new band occurs at different irradiation times, depending on the encapsulation status. Analysis of Veliparib normalized Gaussian areas shows that even after 10 min of UVC exposure, the degradation product is detected, and that area values increase gradually until 120 min of irradiation (Fig. 1(g)). On the other hand, in DPPG + Veliparib samples, Gaussian 5 appears only after 60 min (1 h) of UVC exposure (Fig. 1(g)), thus proving that encapsulation into DPPG liposomes contributes to the delay of the degradation process of Veliparib. Continued UVC exposure led to the disappearance of Gaussian 5, which was verified after 480 min of irradiation (Fig. 1(g)). The final 330 nm electronic excitation band may be attributed to the n–π* transition of the carbonyl group in a linear unsaturated aldehyde.^31^ High absorption above 290 nm, associated with that electronic transition, is pointed to serve as an indication that compounds could be degraded by photolysis.^31^

The results confirm that DPPG liposome encapsulation protected Veliparib from UVC degradation and are in line with PCA analysis (Fig. 1(e)), which highlights degradation differences in the initial irradiation periods. Also, the new absorbance band that emerges around 330 nm in the UV-vis spectra can be associated with a degradation product of Veliparib's carbonyl group. However, to determine the degradation process and products of Veliparib due to UVC irradiation infrared analysis was conducted.

### Analysis of UV damage on Veliparib *via* UV-vis and FTIR – compound degradation process

**Fig. 2:**
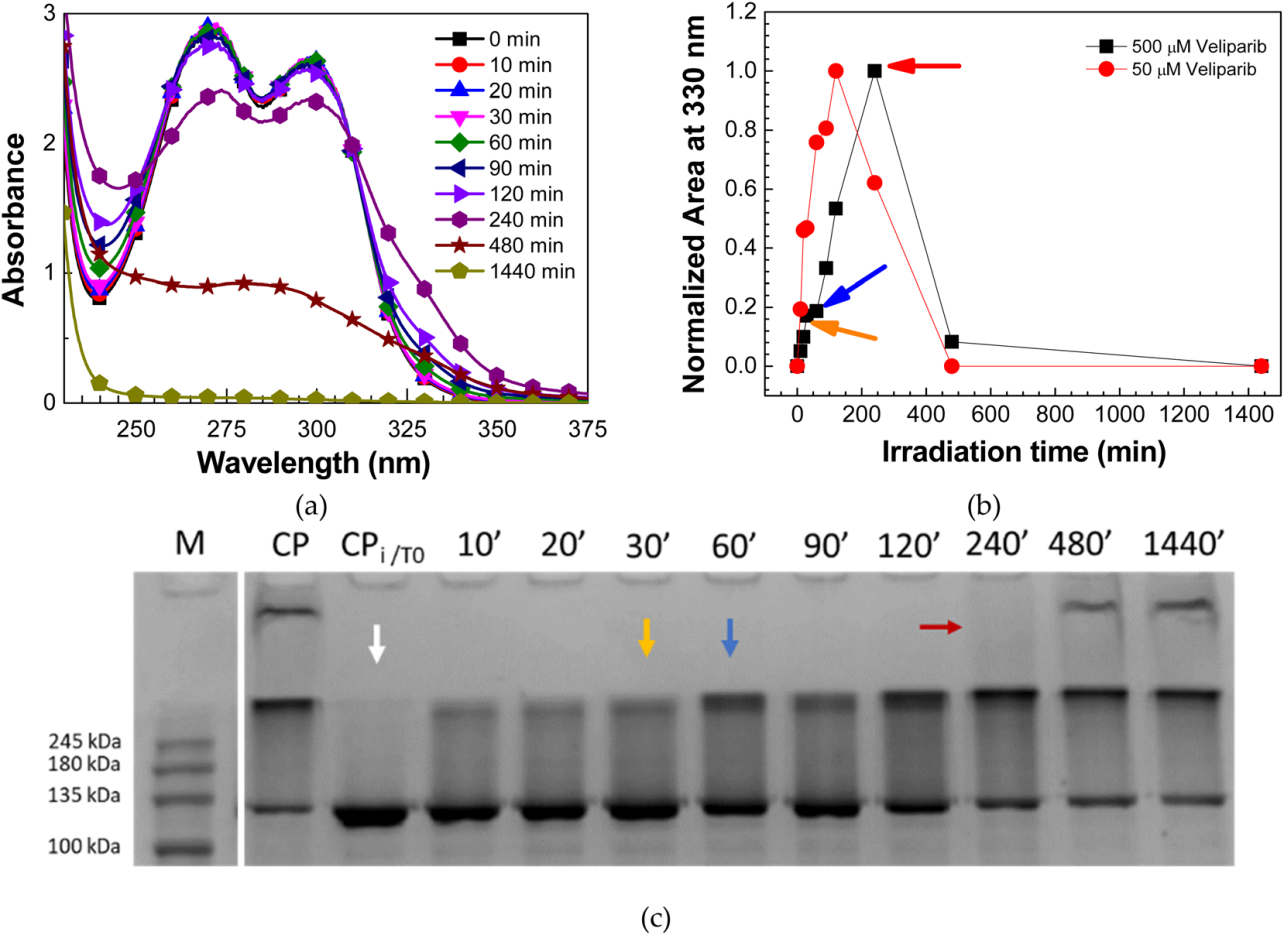
Evaluation of the effect of UVC irradiation on Veliparib's degradation process. (a) UV-vis spectra of 500 µM Veliparib solution at different irradiation periods; (b) comparative analysis of the normalized areas of UV-visible spectra Gaussians 5 (330 nm) of irradiated 50 µM and 500 µM Veliparib samples. In this graph, the lines between points are only guidelines; (c) SDS-PAGE gel analysis of the effect of irradiated Veliparib samples (500 µM) on the auto-modification activity of PARP1. M – NZYColour Protein Marker II (NZYTech); CP – positive control without inhibitor; CPi/T0 – positive control with non-irradiated inhibitor; X’ – Veliparib's irradiation (min). Colored arrows are used to identify the irradiation time points that showcase major alterations in the inhibitory capability of Veliparib and when the normalized area of Gaussian 5 attained a maximum value. These samples were subsequently analyzed by infrared spectroscopy.

To confirm the effect of UVC irradiation on the degradation process and products of Veliparib, a complementary analysis with UV-vis and FTIR spectroscopies was performed. Veliparib samples were used at a final concentration of 500 µM, and UV-visible analysis was employed to follow the degradation process and compare it with the one from the 50 µM samples (Fig. 1(a)). In this way, we want to pinpoint key degradation moments of Veliparib. The degradation process is similar for both samples at 50 µM (Fig. 1(a)) and 500 µM (Fig. 2(a)), however, at higher concentrations, it appears that the process is slower. Since the absorbance values at 270 nm and 295 nm are above 2, with a 500 µM Veliparib sample (Fig. 2(a)), one cannot consider these wavelengths for analysis because of signal saturation. However, the region of interest showcasing the degradation product (330 nm absorbance band), is within the unitary value, thus making the comparative analysis feasible. The normalized areas of Gaussians 5 (330 nm) from 50 µM and 500 µM Veliparib, seen in Fig. 2(b), show that this new band occurs in both samples even after 10 min of irradiation. These results thus state that the appearance of this new band is a direct result of Veliparib exposure to UVC sources. Moreover, one verifies that maximal areas are attained at different irradiation periods, showcasing that the degradation process is slowed down at high concentrations of inhibitor (Fig. 2(b)). This could be due to the reduced number of water molecules and/or photons.

Additionally, irradiated 500 µM Veliparib samples were analyzed for their inhibitory capability on PARP1's enzymatic activity. In this sense, auto-modification activity assays were done following a previously described method.^16^ Results are visualized in an SDS-PAGE gel, where the modified protein presents a shift of PARP1 protein band (114 kDa) to higher weight regions of the gel. In Fig. 2(c), it is confirmed that PARP1 activity was completely inhibited with non-irradiated Veliparib (0 min of UVC exposure), while samples of irradiated inhibitor appear to have lost their inhibitory capability. This loss is perceived by the observation of a shifted protein band to regions above 114 kDa. The increase in UVC exposure led to a progressive loss of Veliparib's inhibitory capability. The effect was maximum after 480 min of UVC exposure (Fig. 2(c)), with the visualization of the almost complete shift of the PARP1 band to regions above 114 kDa. In Fig. 2(b) and (c), we identify irradiation time points that coincide with major alterations in Veliparib's inhibitory capability (yellow, blue, and red arrows) and maximum area of Gaussian 5 (red arrow). These samples were the ones that were used in FTIR analysis to determine the alterations that occurred in the structure of Veliparib and led to the loss of its inhibitory capacity.

**Fig. 3:**
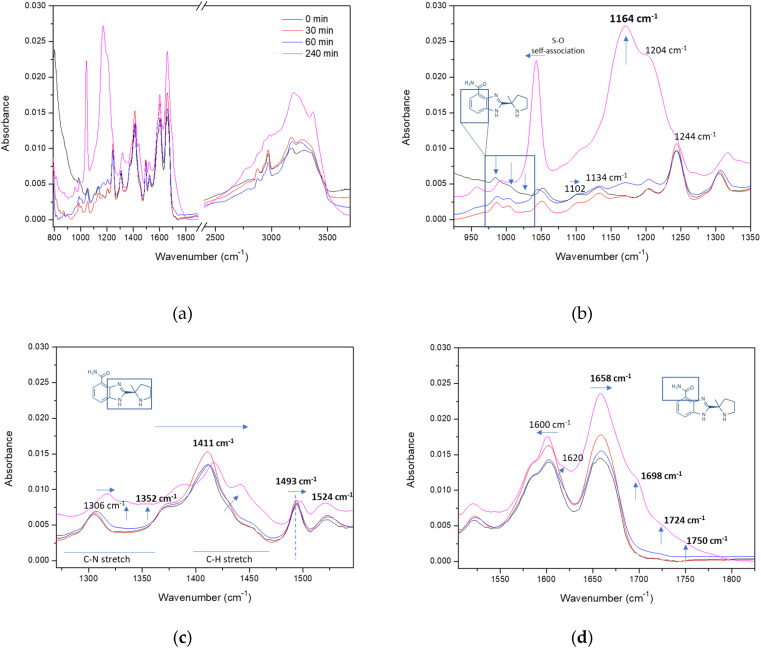
Infrared spectroscopy analysis of UVC irradiated Veliparib samples. (a) Complete FTIR spectra of 500 µM Veliparib at various irradiation periods; (b) FTIR spectra comprising 900 to 1350 cm^−1^ region; (c) FTIR spectra comprising 1275 to 1550 cm^−1^ region; (d) FTIR spectra comprising 1500 to 1825 cm^−1^ region. The code of colors displayed in (a) is the same in all four graphs.

Veliparib samples analyzed by FTIR were irradiated for 30, 60, and 240 min. Also, a non-irradiated sample was used as a control. The Veliparib control sample presents the typical infrared bands that we have assigned previously.^22^ In the irradiated samples, we verified significant alterations in various regions of the spectra (Fig. 3(a)–(d)).

In Fig. 3(b), the absorbance bands at 984, 1004, and 1022 cm^−1^ (identified with blue arrows) present some alteration in their absorbance ratios, with the band at 1022 cm^−1^ not being detected after 30 min of UVC exposure. This band is assigned to the C–C stretch from the cyclohexane ring of Veliparib.^22^ Structural alterations in the benzamide ring of PARPi molecules constitute a severe impact on therapeutic effectiveness of these molecules since it is the core of their inhibitory capability on PARP1. Most PARPi were designed to mimic and compete with the cofactor NAD^+^ for the catalytic domain of PARP1.^7,15,32,33^ They belong to the monoaryl amides and bi-/tri-/tetracyclic lactam compound group,^32^ and their common pharmacophore features are the aromatic ring and carboxamide moiety (benzamide).^34^ Through PARP1's activity assay, it is verified that even after 10 min of irradiation, Veliparib loses some of its inhibitory capability, and that the effect remains the same until 30 min of UVC exposure (Fig. 2(c)). This data and the loss of the cyclohexane vibrational band are coherent and explain one another, thus confirming the UVC effect on the core structure of Veliparib. Additionally, we verify that the bands assigned to the C–N stretch of the aromatic rings (1102, 1134, and 1164 cm^−1^) are also profoundly altered, with bands 1102 cm^−1^ and 1134 cm^−1^ shifted to higher wavenumbers and absorbance of band 1164 cm^−1^ increased significantly (Fig. 3(b)). Maximum absorbance of band 1164 cm^−1^ is observed in the sample irradiated for 240 min. This band is also associated with a secondary amine group,^35^ thus indicating the changes in aromatic rings with nitrogen atoms, specifically the imidazole ring. The increase of absorbance in the 3330–3400 cm^−1^ region points to a possible primary or secondary aliphatic amine (N–H stretch),^35^ thus reinforcing the indication of UVC impact on the aromatic rings with nitrogen.

The vibration band at 1244 cm^−1^, associated with C–C/C–O stretch,^22^ appears diminished with the preceding bands (1164 cm^−1^ and 1204 cm^−1^) after 240 min irradiation, and the band associated with DMSO solvent is shifted to a lower wavenumber when compared to the control samples. This shift is associated with S–O band vibration when DMSO is self-associated.^22,36^

Analysis of the aromatic C–N and C–H stretching vibrations in Fig. 3(c) indicates a shift to higher wavenumbers and the appearance of a new band at 1352 cm^−1^. This band could be assigned to a C–C stretch, a C–N stretch from a secondary aromatic amine, or a C–H bend from a methine functional group.^35^ The latter could result from a possible opening of the benzamide ring, which we verified by the loss of the cyclohexane vibration band in Fig. 3(b). DMSO vibrational bands (1375 and 1410 cm^−1^) and the band associated with Veliparib's C–H asymmetric bend of methyl (–CH_3_) functional group (1438 cm^−1^),^22^ are shifted to higher wavenumbers, with the last presenting an increase in absorbance. This increase can be associated with the degradation process of the aromatic rings. However, the maintenance of C=C–C aromatic stretch bonding (1493 cm^−1^) may indicate that some aromatic species are maintained even after 240 min of UVC irradiation. The same can be inferred with the maintenance of the 1524 cm^−1^ band, previously assigned to aromatic amine CH2 scissoring/C–N and C–C ring stretch.^22^

Finally, the spectral region associated with the benzamide functional groups (amine and carbonyl) in Fig. 3(d) indicates that these groups undergo an additional degree of structural alteration. The vibrational band at 1600 cm^−1^, assigned to –NH_2_ scissoring, is slightly shifted to lower wavenumbers, and a shoulder band surfaces after 240 min of irradiation. The latter appears at 1620 cm^−1^ and is associated with –NH_2_^+^ deformation vibrations.^37^ This shows how the amine functional group is affected by UVC exposure. Additionally, the band assigned to the carboxyl functional group is slightly shifted, and three new vibrational bands appear at higher wavenumbers. In Fig. 3(d), the new band at 1724 cm^−1^ arises only after 60 min of UVC exposure and is assigned to a *cis*-conjugated C=O functional group, while the canonical C=O of Veliparib (1658 cm^−1^) is assigned to a trans conjugated form.^38^ Additionally, it is interesting to notice that the rise of the carbonyl isomer coincides with one of the irradiation periods that shows major alterations in the shift pattern of PARP1's activity assay, Fig. 2(c). This led us to infer that the isomer form leads to a reduction in the inhibitory capability of Veliparib.

The new vibrational band at 1698 cm^−1^, if taken into consideration with the conjugated double bond band, points to a carbonyl group from a conjugated ketone.^35^ This band only appears after 240 min of UVC exposure, and its appearance is further reinforced by the degradation of the cyclohexane ring and points to a putative degradation product resultant from the aromatic ring opening. Additionally, the band that is detected at 1750 cm^−1^ reveals another degradation product, with this vibrational band being associated with an aldehyde or an ester functional group.^35^ Interestingly, this data is coherent with the assignment of the 330 nm UV-vis band, thus showing that UVC has a profound effect on the benzamide ring and the carbonyl functional group. Moreover, we verify that the pharmacophore of Veliparib is readily affected by UVC irradiation, leading to its degradation and the formation of a linear unsaturated aldehyde structure. Moreover, the degradation process points to the loss of double bonds in the imidazole ring and its saturation with hydrogens, which is also perceived by the increase of absorbance in the N–H stretch spectra region (>3200 cm^−1^).

**Fig. 4:**
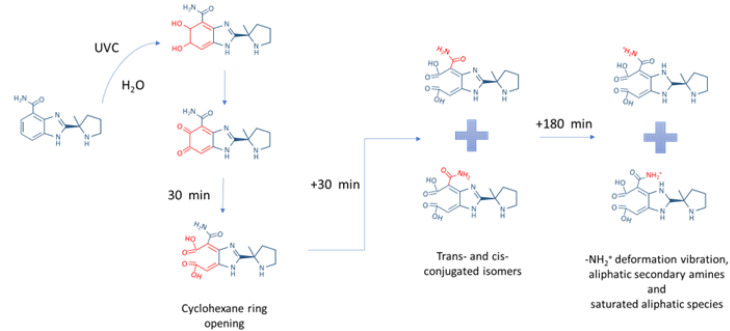
Scheme illustrating the proposed possible degradation process and products of irradiated Veliparib, considering FTIR and UV-vis analysis.

Considering the achieved data, one can propose in Fig. 4, a putative degradation scheme for 500 µM Veliparib due to UVC irradiation. In this scheme, after 30 min of irradiation, the cyclohexane ring becomes an open structure and, upon further irradiation time, a new isomer arises. After 240 min of UVC irradiation, the imidazole ring becomes an aliphatic secondary amine, and the protonation and deformation of the amine functional group in the benzamide are verified. Moreover, the presence of multiple C=O vibrational bands in the 240-minutes spectra suggests that the amine group may be removed from the open benzamide structure, leading to the formation of aldehyde and conjugated ketone species as degradation products.

Complementing this analysis with the data from the previous section, we conclude that the protection that is conferred against UVC degradation through the encapsulation of Veliparib in DPPG liposomes is mainly associated with the benzamide ring and its functional groups. The DPPG protection against degradation is shown to be related to the delay in the growth of the absorbance band centered at 330 nm associated with the degradation, which starts to appear after 30 min of UVC exposure (Fig. 1(e) and (f)). This absorption peak can be associated with benzene ring-opening reactions and the surface of a linear unsaturated aldehyde degradation product. The functionalization of the DPPG liposome surface could further increase Veliparib's protection against irradiation. Coatings such as polymers (*e.g.*, PEG), polyelectrolytes, or even conjugation with other systems could be employed.^39–41^ In particular, PEGylation is a well-established approach to improve liposome stability by reducing aggregation, limiting premature degradation, and increasing circulation time through steric stabilization. Moreover, PEG chain length and surface density can influence the protective effects, as well as alternative surface modifications (*e.g.*, polymer or ligand coatings) that may further enhance liposome robustness.

## Experimental

### Chemicals

1,2-Dipalmitoyl-*sn*-glycero-3-phospho-rac-(1′-glycerol) sodium salt (DPPG) (MW 744.96 g mol^−1^) was purchased from AvantiPolar Lipids (Alabaster, AL, USA), and PARP1 inhibitor Veliparib (ABT-888) (MW 244.3 g mol^−1^) was purchased from AdooQ® Bioscience (Irvine, CA, USA). Veliparib stock solutions (100 mM) were prepared in DMSO anhydrous (99.9%) (Sigma-Aldrich, Missouri, USA).

### Liposome preparation

The DPPG liposomes were prepared following the procedure described in ref. 22. The inhibitor was added to the organic solvent mixture (chloroform and methanol 4 : 1 (v/v) mixture) of 1 mM DPPG. The lipid formulations were subjected to a stream of nitrogen and left under a vacuum in a desiccator for 18 hours. Lipid films were hydrated for 2 h at 47 °C, with Milli-Q ultrapure water (Millipore, Burlington, MA, USA) and subjected to 20 sonication cycles of 30 seconds and 1-minute intervals (tip sonicator UP200S (200 W, 24 kHz) Hielscher Ultrasonics, GmbH, Teltow, Germany), to obtain small unilamellar vesicles. Emulsions were prepared with a Veliparib concentration of 200 µM. Not encapsulated Veliparib molecules were removed by dialysis (Spectra/Por® 4 Dry Standard RC membrane, MWCO 12–14 kDa, Spectrum Labs, Biotech, San Fran-cisco, CA, USA) for 48 h at 4 °C. Encapsulation efficiencies of 50% were attained and were estimated by calculation of the ratio between the absorbance at 275 nm of DPPG + Veliparib before and after dialysis, as previously described.^22^ The specific methodologies for determining encapsulation efficiency, how it varies with different Veliparib concentrations, and the size particle distribution parameters were described in ref. 22.

### Irradiation experiment

Irradiation assessments were performed using aqueous solutions of 50 µM and 500 µM Veliparib, and vesicle suspension of 0.5 mM DPPG and 0.5 mM DPPG + 50 µM Veliparib. Samples were placed in quartz cuvettes and irradiated with a 254 nm UVC germicide lamp (Philips TUV PL-S 5 W/2 P 1CT) at a radiance of 28.9 W m^−2^. The lamp (67 mm bulb) was installed in a custom-made sealed box where the irradiations were also performed. The UVC (254 nm) dose ranged up to 2.5 MJ m^−2^. Samples were analyzed by UV-visible (UV-vis) and Fourier transform infrared (FTIR) spectroscopies. Three independent replicates were used per assay.

### Spectral measurements

UV-visible and FTIR spectra were used to assess drug encapsulation and irradiation effect. Spectra were recorded with a Shimadzu UV-1800 UV/Visible Scanning Spectrophotometer (Kyoto, Kyoto, Japan) and a Bruker IFS 66/S (Billerica, MA, USA) spectrometer in absorbance mode, respectively. FTIR spectra were measured in the 500 to 4000 cm^−1^ wavenumber range with a resolution of 4 cm^−1^, and a total of 128 scans were recorded. Films of liposomes and inhibitors were deposited onto CaF2 supports by water evaporation in vacuum conditions (with a desiccator). CaF_2_ clean support was used as a blank for all sample spectra. Three independent replicates were analyzed per assay.

Principal component analysis (PCA) was performed on UV-visible spectra collected at different irradiation times. This approach reduced the data dimensionality and generated a set of orthogonal components, enabling the identification and interpretation of spectral patterns associated with different irradiation periods.

### Human PARP1 gene cloning, protein expression, and purification

Human PARP1 protein was obtained following the previously described protocol.^42^ Purified protein was concentrated to approximately 1 mg mL^−1^ using an Amicon® Ultra-4 (10 kDa MWCO) (Merck Millipore, Germany), flash frozen in liquid nitrogen, and stored at −80 ᵒC. The storage buffer composition was 50 mM Tris–HCl pH 8.0, 400–500 mM NaCl, 0.1 mM TCEP, and 1 mM EDTA.

### PARP1 auto-modification activity assay

PARP1 auto-modification activity reactions were done following the Langelier protocol^16^ and our previously described adaptation.^42^ Overall, activity reaction used 1 µM protein, 1 µM blunt non-labeled DNA template (Seq. Fw: 5′-AGTACGGTCATCGCG-3′ and Seq. Rv: 5′-CGCGATGACCGTACT-3′), 5 mM beta – nicotinamide adenine dinucleotide (β-NAD+) and incubation buffer (20 mM Tris–HCl pH 7.5, 50 mM NaCl, 5 mM MgCl2). Incubations were done for 10 min at room temperature and halted with SDS-loading buffer (15.6 mM Tris, 100 mM EDTA, 2.5% glycerol, 0.2 M β-mercaptoethanol, 0.26% SDS, 0.001% bromophenol blue). Veliparib (500 µM) inhibitory capability was assessed by adding irradiated samples at 1 µM to the auto-modification reactions. Results were analyzed in 10% SDS-PAGE gel, stained with BlueSafe (NZYTech, Lisbon, Portugal), and recorded in the BioRAD Gel Doc EZ System (Hercules, CA, USA).

## Conclusions

As shower of particles or radiation with UV and lower energies can be created during radiotherapy treatments, the effect of UVC radiation on Veliparib molecules, either encapsulated or not encapsulated, was analyzed, as well as the degradation process of the inhibitor.

Results demonstrated that Veliparib is sensitive to UVC irradiation. The exposure to light leads to compound degradation and loss of inhibitory capability on PARP1 automodification activity. Furthermore, DPPG encapsulation protects Veliparib from UVC irradiation until 30 min of exposure since the absorbance peak centered at 330 nm and associated with degradation only starts to appear after 30 minutes of irradiation. The protective effect of Veliparib is mainly associated with the benzene ring and the delay in the appearance of a linear unsaturated aldehyde degradation product. It was also verified that even though the degradation process appeared to slow down at high concentrations of Veliparib, the surfacing of the band at 330 nm was a result of UVC exposure.

Infrared analysis revealed that the surfacing of the UV-vis 330 nm absorbance band is associated with the degradation of the benzamide ring and the carbonyl functional group. The latter presents several C=O vibrational bands which are associated with isomerization and degradation of the functional group. Through the analysis of PARP1's auto-modification activity, we verified that these structural alterations were responsible for major losses in Veliparib's inhibitory capability.

Moreover, based on the infrared results, we propose a putative UVC-induced degradation process, and possible degradation products, such as aldehyde and conjugated ketone structures that arise from the degradation of the benzamide pharmacophore, have been identified. In this way, the UVC degradation effect on Veliparib and the protective effect given by the encapsulation of this compound in DPPG liposomes was confirmed. This allows us to infer that some drawbacks associated with conventional PARPi therapy, such as loss of therapeutic efficacy, can be circumvented by liposome nano-systems encapsulating these inhibitors. This work serves as a proof-of-concept on the degradation effect of UVC radiation on PARPi and fills a gap in PARP1 inhibitor therapy knowledge while representing the first report of its kind regarding the low-energy radiation effect on PARPi. However, to completely understand the combined radiation effect and PARPi therapy in cancer treatment, cell-based assays should be implemented to assess how the results depicted here are translated into normal and cancer cell models and how degradation products can affect cell viability. This consideration extends beyond the mere loss of therapeutic efficacy to include the intrinsic effects of the degradation products themselves. Our preliminary cytotoxicity studies of free Veliparib and liposome-encapsulated Veliparib in squamous cell carcinoma cells (MET-1) and non-cancerous keratinocytes (HaCaT) indicate that the free drug exhibits greater toxicity than its encapsulated form. In MET-1 cells, the preliminary IC_50_ values were 80 µM for free Veliparib and 160 µM for the encapsulated formulation. In HaCaT cells, the corresponding IC_50_ values were 160 µM and 320 µM, respectively. Therefore, only through a more comprehensive evaluation of the effects of PARP inhibitors—whether encapsulated or not—across different cell types, together with phototoxicity studies under UV and lower-energy radiation, can cancer treatment with PARP inhibitors be optimized.

## Author contributions

Conceptualization, C. C., and M. R.; methodology, C. C. and M. R.; validation, C. C., and M. R.; formal analysis, C. C., and M. R.; investigation, C. C., E. M., and M. R.; resources, E. M., P. A. R., and M. R.; data curation, C. C., and M. R.; writing—original draft preparation, C. C.; writing—review and editing, C. C., E. M., P. A. R., and M. R.; visualization, C. C.; supervision, E. M., P. A. R., and M. R.; project administration, E. M., P. A. R., and M. R.; funding acquisition, E. M., P. A. R., and M. R. All authors have read and agreed to the published version of the manuscript.

## Conflicts of interest

There are no conflicts to declare.

## Appendix

**Fig. 5:**
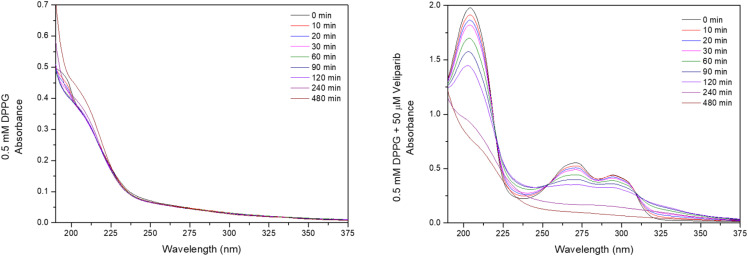
DPPG and DPPG encapsulating Veliparib irradiation assay with UVC lamp. Absorbance spectra of (a) 0.5 mM DPPG formulation; and (b) 0.5 mM DPPG + 50 µM Veliparib for different irradiation times.

**Fig. 6:**
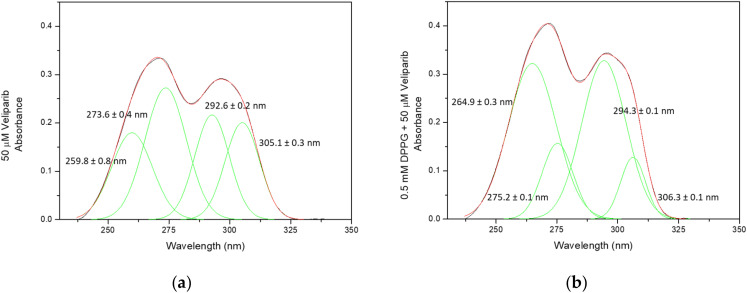
Gaussian analysis of Veliparib and DPPG + Veliparib samples. Data reveals that Veliparib spectra comprise four Gaussians and that after drug encapsulation in DPPG liposomes, these Gaussians present a red shift on wavelength number. (a) UV-vis spectra of 50 µM Veliparib; (b) UV-vis spectra of 0.5 mM DPPG + 50 µM Veliparib.

**Table 2:** Analysis of Veliparib and DPPG + Veliparib wavelength shift of Gaussian 2 and 5 upon UVC irradiation

Irradiation time (min)	Veliparib peak Position^a^ (nm)	DPPG + Veliparib peak position^a^ (nm)	Veliparib peak Position^b^ (nm)	DPPG + Veliparib peak position^b^ (nm)
0	273.7 ± 0.4	275.3 ± 0.1	n.d.	n.d.
10	273.9 ± 0.3	274.9 ± 0.2	325.2 ± 0.8	325.2 ± 0.8
20	273.5 ± 0.2	274.5 ± 0.1	325.4 ± 0.7	325.4 ± 0.7
30	273.4 ± 0.2	274.1 ± 0.2	327.1 ± 0.4	327.1 ± 0.4
60	273.04 ± 0.32	273.8 ± 0.3	327.8 ± 0.5	327.8 ± 0.5
90	273.4 ± 0.2	273.7 ± 0.2	328.5 ± 0.5	328.5 ± 0.5
120	274.1 ± 0.3	273.9 ± 0.2	328.1 ± 0.7	328.1 ± 0.7
240	275.2 ± 0.5	278.7 ± 0.6	330.3 ± 0.9	330.3 ± 0.9
480	n.d.	n.d.	n.d.	n.d.

a Peak position of Guassian 2.

b Peak position of Guassian 5 n.d. means not detected.

## Supplementary Material

RA-016-D5RA02652K-s001

RA-016-D5RA02652K-s002

## Data Availability

The data supporting this article have been included as part of the supplementary information (SI) (see Appendix A). Further inquiries can be directed to the corresponding author. The raw data supporting the conclusions of this article will be made available by the authors on request. Supplementary information is available. See DOI: https://doi.org/10.1039/d5ra02652k.
