# Supplementary figures and images for: Enhancing Veliparib PARP1 inhibitor stability against UVC degradation via DPPG liposome encapsulation
Source: RSC Adv. 2026 Mar 17;16(16):14676–87. doi: 10.1039/d5ra02652k (PMC12994379; doi:10.1039/d5ra02652k)

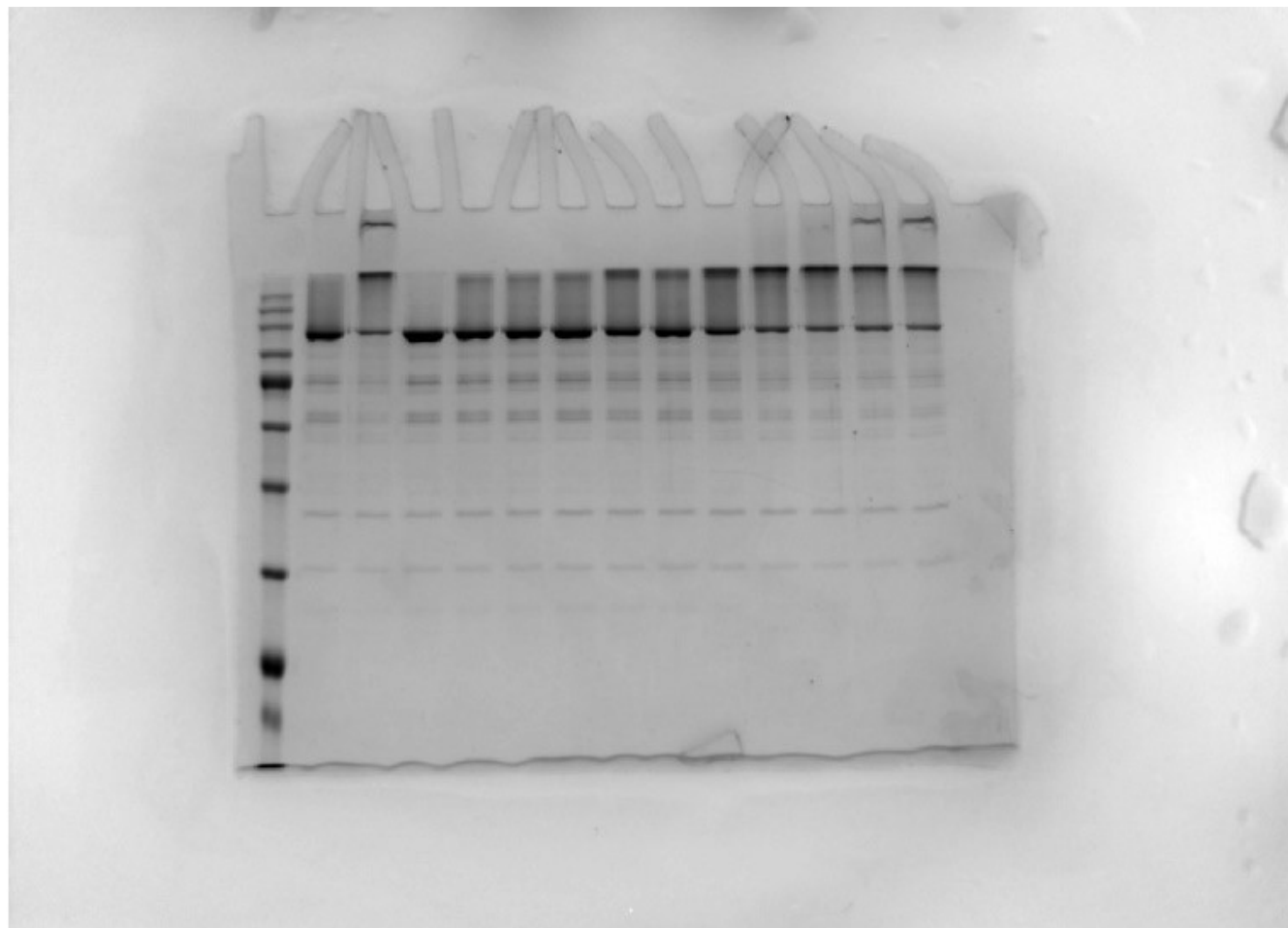

Supplement: RA-016-D5RA02652K-s001 [file RA-016-D5RA02652K-s001.pdf]
