# Supplementary material for: Enhancing Veliparib PARP1 inhibitor stability against UVC degradation via DPPG liposome encapsulation
Source: RSC Adv. 2026 Mar 17;16(16):14676–87. doi: 10.1039/d5ra02652k (PMC12994379; doi:10.1039/d5ra02652k)
Supplement: RA-016-D5RA02652K-s002 [file RA-016-D5RA02652K-s002.pdf]

Supplementary Information (SI) for RSC Advances.
This journal is © The Royal Society of Chemistry 2026

Appendix A

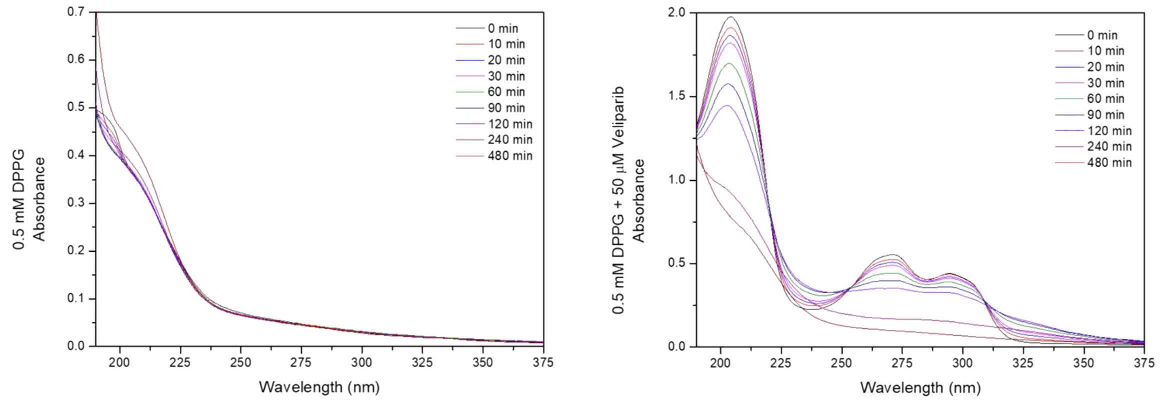

(a) (b)

Figure A1. DPPG and DPPG encapsulating Veliparib irradiation assay with UVC lamp. Absorbance spectra of (a) 0.5 mM DPPG formulation; and (b) 0.5 mM DPPG + 50 μM Veliparib for different irradiation times.

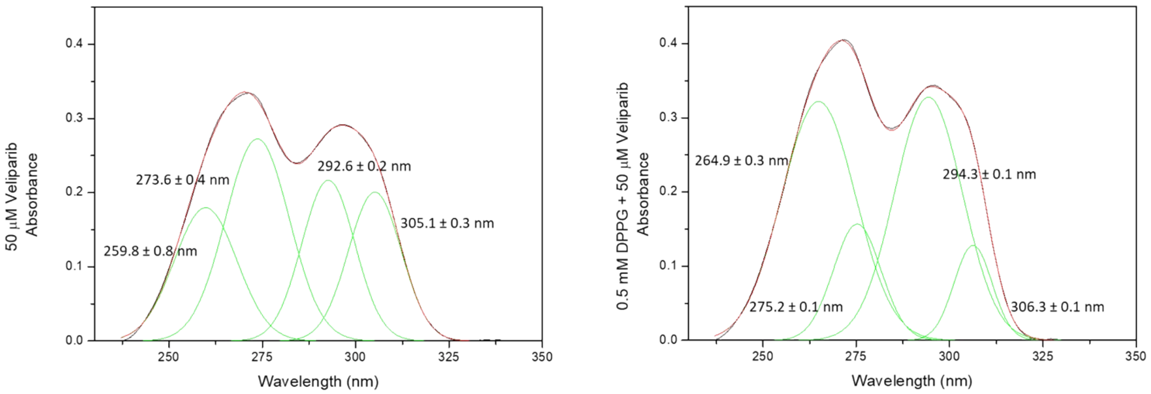

(a) (b)

Figure A2. Gaussian analysis of Veliparib and DPPG+Veliparib samples. Data reveals that Veliparib spectra comprise four Gaussians and that after drug encapsulation in DPPG liposomes, these Gaussians present a red shift on wavelength number. (a) UV-vis spectra of 50 μM Veliparib; (b) UV-vis spectra of 0.5 mM DPPG + 50 μM Veliparib.

Table A1. Analysis of Veliparib and DPPG + Veliparib wavelength shift of Gaussian 2 and 5 upon UVC irradiation.

Irradiation Time (min)	Veliparib Peak Position[1](nm)	DPPG+Veliparib Peak Position[1] (nm)	Veliparib Peak Position[2](nm)	DPPG+Veliparib Peak Position[2] (nm)
0	273.7 ± 0.4	275.3 ± 0.1	n.d.	n.d.
10	273.9 ± 0.3	274.9 ± 0.2	325.2 ± 0.8	325.2 ± 0.8
20	273.5 ± 0.2	274.5 ± 0.1	325.4 ± 0.7	325.4 ± 0.7
30	273.4 ± 0.2	274.1 ± 0.2	327.1 ± 0.4	327.1 ± 0.4
60	273.04 ± 0.32	273.8 ± 0.3	327.8 ± 0.5	327.8 ± 0.5
90	273.4 ± 0.2	273.7 ± 0.2	328.5 ± 0.5	328.5 ± 0.5
120	274.1 ± 0.3	273.9 ± 0.2	328.1 ± 0.7	328.1 ± 0.7
240	275.2 ± 0.5	278.7 ± 0.6	330.3 ± 0.9	330.3 ± 0.9
480	n.d.	n.d.	n.d.	n.d.

[1]Peak position of Guassian 2. [2]Peak position of Guassian 5.n.d. means not detected.
